# Female smokers beyond the perimenopausal period are at increased risk of chronic obstructive pulmonary disease: a systematic review and meta-analysis

**DOI:** 10.1186/1465-9921-7-52

**Published:** 2006-03-29

**Authors:** Wen Qi Gan, SF Paul Man, Dirkje S Postma, Patricia Camp, Don D Sin

**Affiliations:** 1 James Hogg iCAPTURE Center for Cardiovascular and Respiratory Research, University of British Columbia, Vancouver, B.C., Canada; 2 Department of Medicine (Pulmonary Division), University of British Columbia, Vancouver, B.C., Canada; 3 Department of Pulmonology, University Hospital, University of Groningen, Groningen, The Netherlands

## Abstract

**Background:**

Recent reports indicate that over the next decade rates of chronic obstructive pulmonary disease (COPD) in women will exceed those in men in the western world, though in most jurisdictions, women continue to smoke less compared with men. Whether female adult smokers are biologically more susceptible to COPD is unknown. This study reviewed the available evidence to determine whether female adult smokers have a faster decline in forced expiratory volume in one second (FEV_1_) compared with male adult smokers and whether age modifies the relationship between cigarette smoke and lung function decline.

**Methods:**

A systematic review and a meta-analysis was performed of population-based cohort studies that had a follow-up period of at least 3 years, measured FEV_1 _on at least two different time points, and presented FEV_1 _data stratified by gender and smoking status in adults.

**Results:**

Of the 646 potentially relevant articles, 11 studies met these criteria and were included in the analyses (N = 55 709 participants). There was heterogeneity in gender-related results across the studies. However, on average current smokers had a faster annual decline rate in FEV_1_% predicted compared with never and former smokers. Female current smokers had with increasing age a significantly faster annual decline in FEV_1_% predicted than male current smokers (linear regression analysis, R^2 ^= 0.56; p = 0.008). Age did not materially affect the rate of decline in FEV_1_% predicted in male and female former and never smokers (p = 0.775 and p = 0.326, respectively).

**Conclusion:**

As female smokers age, they appear to experience an accelerated decline in FEV_1_% predicted compared with male smokers. Future research powered specifically on gender-related changes in lung function is needed to confirm these early findings.

## Background

Chronic obstructive pulmonary disease (COPD) is a major cause of death in North America and Europe and the only major disease for which the morbidity and mortality are still increasing in these continents [1,2]. Although COPD is currently the 4th-leading cause of mortality and the 12th-leading cause of disability, by the year 2020 it will be the 3rd-leading cause of death and the 5th-leading cause of disability worldwide [3,4]. Strikingly, this projected increase in COPD-related morbidity and mortality will be driven largely by the female population, a trend that started 20 years ago [5]. Some have ascribed this trend to increased smoking rates in women over the past two decades [6]. However, there are likely to be other factors involved. While female smoking rates have indeed increased relative to male rates since the 1970's, female smoking rates continue to be lower than those for men [5,7]. For example, in the US in 2003, 19% of adult women smoked versus 24% of adult men [8]. Moreover, even when women smoke, they consume on average fewer cigarettes per day and have lower serum cotinine levels compared with men, indicating that cigarette smoke exposure *per se *cannot account for the rising COPD burden in women [9]. These data raise the possibility that female smokers may be biologically more susceptible to COPD compared to male smokers. We conducted a systematic review and a meta-analysis to determine whether female smokers do or do not have increased susceptibility to COPD compared with male smokers. Additionally, since age is a major determinant of changes in lung function [10], we sought to determine whether age modified the relationship between smoking and lung function decline in both men and women.

## Methods

### Search for relevant studies

Using PUBMED (1966–January 2006) and EMBASE (1980–January 2006) electronic databases, we conducted a comprehensive literature search to identify studies related to the decline of lung function published before January 2006. We used lung function sensitive terms (forced expiratory volume, vital capacity) combined with design sensitive terms (cohort studies, longitudinal studies, follow-up studies, prospective studies), and smoking sensitive terms (smoke, cigarette, smoking) in our searches. The electronic searches were supplemented by scanning of the reference lists from retrieved articles to identify additional studies that may have been missed during the electronic search. We also contacted the primary authors of retrieved studies for additional data and/or clarification of data, where necessary.

### Study selection and data abstraction

The primary objective of this study was to compare the annual decline of lung function, measured as percent predicted forced expiratory volume in one second (FEV_1 _% pred), which is an important phenotype of COPD [11], between men and women stratified according to smoking status. To mitigate methodological biases, we limited our search to studies that: (1) were population-based; (2) employed a longitudinal cohort design; (3) had a follow-up of at least 3 years; (4) measured FEV_1 _on at least two different time points; and (5) presented FEV_1 _data stratified by gender and smoking status. We excluded cross-sectional studies, or studies that evaluated occupational exposures on lung function. We also excluded studies whose primary focus was on secondhand smoke exposures. From each retrieved article, two independent investigators abstracted the following information: project name, sample size, average age at baseline, proportionality of women, duration of follow up, and annual decline rate of FEV_1 _% pred stratified by gender and smoking status (Table 1, Table 2). Any questions or discrepancies regarding these data were resolved through iteration and consensus.

**Table 1:** Characteristics of studies included in meta-analyses*

**Source**	**Project name**	**Sample size**	**Women (%)**	**Average age at baseline (year)**	**Duration of follow up (year)**
Viegi et al,^22 ^2001	Po River Delta Epidemiologic Study, North Italy	1774	51	32	8
Chinn et al,^12 ^2005	European Community Respiratory Health Survey II, 27 centers, 26 were in western Europe and one was in the USA	6654	51	34	9
Rijcken et al,^13 ^1995	Vlagtwedde-Vlaardingen study in the Netherlands	1619	43	39	25
Jedrychowski et al,^14 ^1986	Cracow Study in Cracow, Poland	1364	64	40	13
James et al,^15 ^2005	Busselton Health Study in Busselton, Western Australia	9317	51	42	29
Tashkin et al,^16 ^1984	UCLA Population Studies in Los Angeles County, USA	2401	54	46	5
Sherrill et al,^17 ^1996	Tucson Epidemiology Study of Obstructive Lung Disease in Tucson, Arizona, USA	477	41	48	8
Connett et al,^23 ^2003^†^	Lung Health Study, 10 centres, nine in the USA, one in Canada	5346	37	48	5
Xu et al,^18 ^1992	Six Cities Study in the USA	12 080	55	49	6
Vestbo et al,^19 ^1996	Copenhagen City Heart Study, Denmark	9435	57	53	5
Griffith et al,^20 ^2001	Cardiovascular Health Study in the USA	5242	57	73	7

Symbols: *: Order in table: average age at baseline; †: The participants were smokers with mild-to-moderate COPD.

**Table 2:** Annual decline rate in FEV_1_% pred/yr in men and women according to smoking status

**Source**	**Average age at baseline (year)**	**Never smokers**			**Former smokers**			**Current smokers**		
				
		**Women**	**Men**	Difference*	**Women**	**Men**	Difference*	**Women**	**Men**	Difference*
Viegi et al,^22 ^2001	32	NA	NA	NA	-0.12	-0.21	0.09	0.12	0.13	-0.01
C hinn et al,^12 ^2005	34	0.78	0.76	0.02	0.91	0.76	0.15	0.88	0.84	0.04
Rijcken et al,^13 ^1995	39	0.83	0.96	-0.13	0.89	0.87	0.02	0.97	1.11	-0.14
Jedrychow ski et al,^14 ^1986	40	1.35	1.13	0.22	NA	NA	NA	1.41	1.46	-0.05
James et al,^15 ^2005	42	0.87	0.91	-0.04	0.99	1.01	-0.02	1.05	1.22	-0.17
Tashkin et al,^16 ^1984	46	1.51	1.70	-0.19	1.36	1.65	-0.29	1.97	2.15	-0.18
Sherrill et al,^17 ^1996	48	0.50	0.44	0.06	0.49	0.85	-0.36	0.66	0.49	0.17
Connett et al,^23 ^2003	48	NA	NA	NA	0.37	0.07	0.30	1.20	1.05	0.15
Xu et al,^18 ^1992	49	1.08	0.98	0.10	1.11	0.89	0.22	1.42	1.37	0.05

Each cell represents annual change in FEV_1_% pred/yr, unless otherwise indicated.

Symbols: *:A positive number denotes a larger decline in FEV_1_% pred in women; a negative number denotes a large decline in FEV_1_% pred in men; †: Never smokers and former smokers were combined as non-smokers in the article since they did not differ in FEV_1_% pred decline.

### Statistical analysis

We used the annual change in the rate of FEV_1_% pred reported in the studies to conduct the primary analyses. Annual changes in FEV_1_% pred were calculated by subtracting the final FEV_1_% pred from the baseline value and dividing the difference by the number of years of follow-up. For studies that only provided absolute FEV_1 _values [12-20], we calculated FEV_1_% pred by applying a published prediction equation to the absolute values [21]. The reported baseline mean age and height were used in these calculations. For studies that did not report data on the subjects' height [12,14,17-19], we imputed 174 cm for men and 161 cm for women because the populations of these studies had similar race and age profiles as those reported in James's study (Table 2) [15]. We compared the annual changes in FEV_1_% pred between women and men across smoking status by using male values as the referent. A positive value denoted a larger decline in women, while a negative value denoted a larger decline in men. We hypothesized that age might be an important modifier for the relationship between smoking and gender-related decline in lung function since the incidence of obstructive airways disease in women increases sharply in the postmenopausal period [5]. We used both unweighted and weighted linear regression techniques to assess gender-related differences in the annual decline of FEV_1_% pred. In the weighted analysis, we used the sample size of men and women in each smoking category as the weights. All tests were two-tailed in nature and were performed using statistical software SAS (version 9.1, SAS Institute, Carey, N.C).

## Results

**Figure 1:**
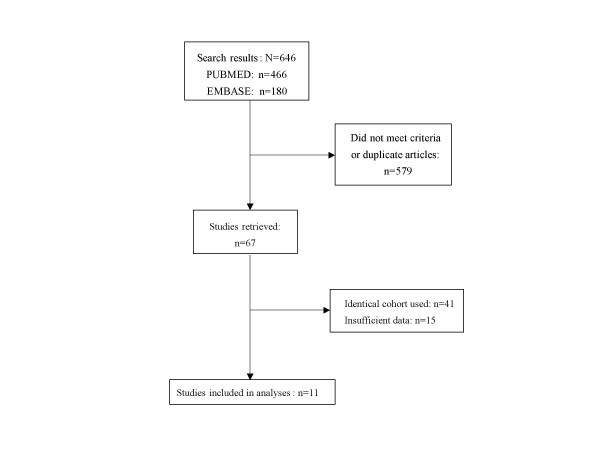
Flow diagram of study selection.

A summary of the search strategy is shown in Figure 1. The original search yielded 466 and 180 citations in PUBMED and EMBASE, respectively. The abstracts of these articles were selected and reviewed. Of these, 67 articles were retrieved for a detailed review. After excluding studies that used identical cohorts (n = 41) and studies that had insufficient data (n = 15), we were left with 11 original studies that met the inclusion criteria. The baseline characteristics of these studies are summarized in Table 1. Collectively, there were 55 709 participants in these studies, 52% were women, and the baseline average age of the cohorts varied from 32 to 73 years. The duration of follow-up ranged from 5 to 29 years.

Table 2 summarizes the annual decline in FEV_1_% pred in both men and women according to smoking status. In general, older cohorts experienced a faster decline in FEV_1_% pred/yr compared with younger cohorts and current smokers had a faster decline in FEV_1_% pred/yr compared with never smokers. Former smokers had similar decline rates in FEV_1_% pred/yr as never smokers. There were four studies that provided data on lung function changes stratified by the mean daily consumption of cigarettes [15,18,19,22]. There was a dose-dependent acceleration in the decline of FEV_1_% pred/yr (Table 3).

**Table 3:** Annual decline rate in FEV_1_% pred/yr for female and male current smokers stratified by the daily amount of cigarette consumption

**Source**	**Average age at baseline (year)**	**Never smokers**			**< 15 g/day**			**15 g/day**		
		
		**Women**	**Men**	Difference*	**Women**	**Men**	Difference*	**Women**	**Men**	Difference*
Viegi et al,^22 ^2001	32	NA	NA	NA	0.08	0.12	-0.04	0.22	0.15	0.07
James et al,^15 ^2005	42	0.87	0.91	-0.04	0.97	1.12	-0.15	1.13	1.26	-0.13
Xu et al,^18 ^1992	49	1.08	0.98	0.10	1.16	0.97	0.19	1.51	1.44	0.07
Vestbo et al,^1 ^1996	53	1.00	0.83	0.17	1.23	0.98	0.25	1.32	1.22	0.10
Total	--	0.99	0.91	0.08	1.10	0.95	0.15	1.35	1.23	0.12

Each cell represents annual change in FEV_1_% pred, unless otherwise indicated.

Symbols: *: A positive number denotes a larger decline in FEV_1_% pred in women; a negative number denotes a larger decline in FEV_1_% pred in men.

**Figure 2:**
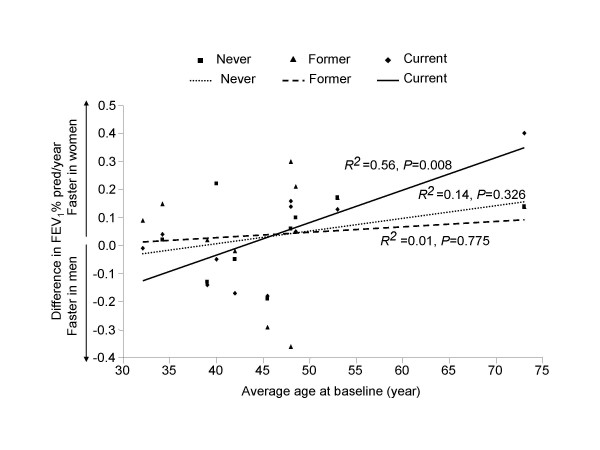
**Unweighted analysis of the relationship between age and gender-related differences in the annual decline in FEV_1_% pred according to smoking status **Abbreviation: FEV_1_: forced expiratory volume in one second.

**Figure 3:**
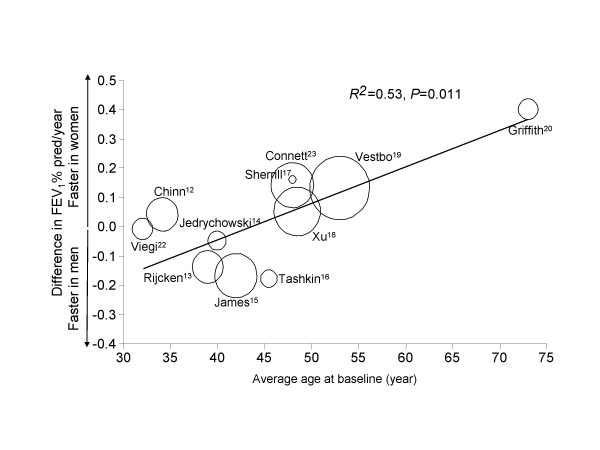
**Weighted analysis of the relationship between age and gender-related differences in the annual decline in FEV_1_% pred for current smokers **The regression line is weighted by the numbers of current smokers. The diameter of each circle is proportional to the number of current smokers in each study. Abbreviation: FEV_1_: forced expiratory volume in one second.

In current smokers, with increasing age, women had a significantly faster decline in FEV_1_% pred/yr compared with men (R^2 ^= 0.56; p = 0.008), while in former and never smokers, age did not significantly modify the rate of decline in FEV_1_% pred/yr between men and women (p = 0.775 and p = 0.326, respectively) (Figure 2). There were no material differences in the results between the weighted and unweighted analyses. The three average age-difference in FEV_1_% pred/yr regression lines diverged at ~45 to 50 years of age. As a sensitivity assessment, we repeated the analysis after excluding the study by Griffith and colleagues [20], which appeared to an outlier in Figure 3. In the sensitivity analysis, female compared with male smokers still had a faster decline in FEV_1_% pred/yr (R^2 ^= 0.40; p = 0.050), while in former smokers and never smokers, there were no gender differences (in former smokers, R^2 ^= 0.14; p = 0.323; in never smokers, R^2 ^= 0.28 and p = 0.179).

## Discussion

The present systematic review indicates that female compared with male smokers experienced a faster decline in lung function beyond age 45 to 50 years. This trend was evident even in female smokers who smoked only a modest amount of cigarettes (<15 g/day). In non- or ex-smokers, there were no significant gender-related changes in FEV_1_% pred over time. However, there was considerable heterogeneity in the results across the studies (see table 2 and figure 3) and as such these data should be interpreted cautiously. Additional prospective longitudinal studies powered specifically on gender-related changes in lung function in the post-menopausal age group are needed to confirm these observations.

The findings from the present study are consistent with other studies, which were not included in this review [21-29]. Prescott and colleagues reported similar findings from two independent population samples: Copenhagen City Heart Study (CCHS) and Glostrup Population Studies (GPS) [24]. In both samples, when adjusted for pack-years of smoking, female smokers had a faster decline in lung function compared with male smokers. In the CCHS, the estimated excess loss of FEV_1 _was 7.4 ml per pack-year in female current smokers and 6.3 ml per pack-year in male current smokers. In the GPS, the estimated excess loss of FEV_1 _was 10.5 ml per pack-year in the female current smokers and 8.4 ml per pack-year in the male current smokers. Importantly, in both samples, even after adjustments of daily tobacco consumption and years of smoking, female smokers had a higher risk of hospitalization for COPD compared with male smokers (relative risk, RR, 1.5, 95% confidence interval, CI, 1.2–2.1 in the CCHS and RR, 3.6, 95% CI, 1.4–9.0 in the GPS) [24]. Furthermore, women with impaired lung function (FEV_1 _< 40% pred) had a higher risk of death from all causes (RR, 5.0 for women, 2.7 for men) and of deaths from obstructive lung diseases (RR, 57 for women, 34 for men,) compared with men [25]. Xu and colleagues studied 1 618 male and 1 669 female adults aged 40–69 yrs in the Beijing Respiratory Health Study [28]. Although female never smokers had better lung function than did male never smokers, female current smokers had significantly lower lung function compared with male smokers [28]. In a genetics study of early onset COPD, Silverman and colleagues found that female first-degree current or ex-smoking relatives of the probands were almost two times more likely to demonstrate mild airflow limitation (FEV_1 _<80% predicted) and over three times more likely to have severe airflow limitation (FEV_1 _<40% predicted) than did male relatives [29].

Although the present study did not evaluate effects of smoking cessation on lung function in men and women, data from the Lung Health Study indicates that female quitters may experience larger gains in lung function than do male sustained quitters. In that study, female sustained quitters experienced a 2.5 fold larger improvement in FEV_1_% pred than did male sustained quitters after one year of smoking cessation [30]. These data, in conjunction with results of the present systematic review, suggest that female smokers have increased susceptibility for COPD, especially after age 45 to 50 years. With smoking cessation, however, female quitters may experience a larger recovery of their lung function than do male quitters.

Although our study was not designed to evaluate the effects of smoking in adolescent youths, previous studies indicate that smoking may also have a greater (negative) impact on lung growth in female than male youngsters. Gold et al [31] found that among adolescents, smoking five or more cigarettes a day, as compared with never smokers, was associated with a 1.09% per year reduction in the growth rate of FEV_1 _in girls, while for boys, smoking reduced FEV_1 _growth by only 0.20%/yr. Patel et al [27] found that exposure to cigarette smoke during childhood was an independent risk factor for the development of obstructive airways disease in women but not in men. Thus, the relationship between gender, age and FEV_1 _changes may be U-shaped.

The mechanisms responsible for the increased susceptibility of women to cigarette smoke are largely unknown. There is now a general consensus that inflammation is at the heart of the pathobiology of COPD and that the inflammatory process involves both the lung (airways and parenchyma) and the systemic circulation [32-34]. The intensity of the inflammatory process in the airways and in the systemic circulation is associated with severity of FEV_1 _impairment [33,34]. Whether women are more likely to demonstrate airway inflammation compared with men is unknown. Interestingly, women in the general population are known to have higher circulating C-reactive protein levels, a marker of systemic inflammation, but only after ~50 years of age [35]. Since active smoking amplifies systemic inflammation, independent of other factors [36], smoking-inflammation pathway may be an important contributor to the increased risk observed in women in the peri and post-menopausal periods. Further research is needed to confirm this hypothesis.

Another potential mechanism may relate to bronchial hyperresponsiveness. In the Lung Health Study, there was a higher prevalence of bronchial hyperresponsiveness among women than among men (85% in women versus 59% of the men) [37]. In another population-based study, Leynaert and coworkers demonstrated increased prevalence of bronchial hyperresponsiveness in women, even after adjustments for respiratory symptoms, atopy, or lung function parameters [38]. Paoletti et al [39] also found increased risk of bronchial hyperresponsiveness among women compared with men independent of baseline lung function. In women, they observed that current smokers had significantly more reactive airways than did non- or ex-smokers. However, in men, smoking status made no material impact on bronchial responsiveness [39]. These data may be clinically relevant since bronchial hyperresponsiveness has been associated with increased risk of both COPD progression [40] and COPD mortality [41].

Additionally, cigarette smoke may modify hormonal status in women, which may affect lung function. Women who are active smokers become relatively estrogen deficient compared with non-smokers because cigarette smoke induces cytochrome P450 isoenzymes CYP1A1 and CYP1A2, which alter estrogen metabolism leading to increased production of inactive catechols [42]. Hormone replacement therapy in the post-menopausal period is associated with improved lung function, reducing the risk of airflow obstruction by ~25% [43]. Hormone replacement therapy also reduces bronchial hyperresponsiveness in post-menopausal women [44].

An alternative hypothesis for higher susceptibility of females to smoking may be differences in lung development between females and males. Interestingly, relative to male rates, female rates of obstructive airway diseases increase sharply during adolescence [45]. Before pubescence, girls have smaller lung volumes than do boys but generate higher flows [46]. During teenage years, airways and lung volumes demonstrate isotropic growth in boys. In girls, however, airway growth becomes disproportionately smaller relative to lung volume growth, indicating dysanapsis [47]. Thus, for any given lung volume and size, women have smaller airways compared with men, which may make the airways more susceptible to the adverse effects of cigarette smoke.

There were several limitations to the study. Firstly, we used only a crude marker of smoking (i.e. self-report of smoking). Since male smokers generally smoke more cigarettes than do female smokers and have a longer smoking history, we may have underestimated the true effects of cigarette smoking in the female population [9]. Secondly, as with most systematic reviews, publication bias is a source of concern. Figure 3 indicates that there were no material differences in results between large and small studies, suggesting that publication bias did not significantly affect the results.

## Conclusion

We found that beyond age 45 to 50 years, female smokers appear to experience an accelerated decline in FEV_1_% pred/yr compared with male smokers. Additional prospective longitudinal studies powered specifically on gender-related changes in lung function in the post-menopausal age group are needed to confirm these observations. In view of the growing incidence of smoking and the COPD in the female population, there is an urgent need to promote smoking abstinence and cessation in the female population.

## Abbreviations

CCHS: Copenhagen City Heart Study

COPD: chronic obstructive pulmonary disease

FEV_1_: forced expiratory volume in one second

GPS: Glostrup Population Studies

Pred: predicted

RR: relative risk

Yr: year

## Competing interests

This project is supported by ICEBERGS (Interdisciplinary Capacity Enhancement: Bridging Excellence in Respiratory Disease and Gender Studies), which is funded by the Canadian Institutes of Health Research (IGH / ICRH), the Canadian Lung Association, and the Heart and Stroke Foundation of Canada.

## Authors' contributions

All authors have made substantial intellectual contribution to the interpretation of the results and drafting of the manuscript.
